# How to Maintain Discipline

**Published:** 1877-12

**Authors:** C. A. Logan

**Affiliations:** 99 Ashland Avenue


*To the Editor of the* Journal and Examiner :—

An editorial in No. 363 of the *New York Medical Record*, entitled, "How to Maintain Discipline," has a few suggestive ideas, which might be extended to more specific conclusions with general advantage. As a stranger, recently located in Chicago, the writer of this communication is moved to the expression of some opinions upon the hackneyed and threadbare subject of the Code Medical.

And first, as to the usefulness of a code of ethics, "denied by a late President of the American Medical Association," as stated in the article above referred to.

But few medical men, it would seem, even among those who class the profession as a simple business pursuit, should be willing to blot out the restrictions pertaining to the conduct of the business, or to break down the regulations which shape and govern the intercourse of the merchants engaged in it. No reference need be made here, to those who place the practice of medicine upon a far higher plane, and seek to remove it beyond the atmosphere of commercial influences.

To both classes—or to all classes, if there be others revolving between the orbits of these extremes—a specific understanding as to what shall, and what shall not be considered *en règle*, would seem to be as necessary as the statutory enactment of moral and penal codes for the proper ordering of society, and the protection of the personal rights and safety of its members. Not only are governments built upon codes of law, but the principle underlying their necessity, is extended down to every department of life, involving the idea of similar effort, whether of an associated nature or not. Hence, every corporation, public and private; every guild; every trades-union; every society, whether of a business, religious, benevolent, literary or scientific character, has its prescribed formulæ of enactments, staking out the boundary lines of correct deportment, and running the parallels of conventional rectitude.

The extent to which one might moralize upon this indisputable fact, seems without limit. The hard essence of it is, that men are constructed differently in their mental organizations, reflecting different sentiments according to the nature and balance of the mind which conceives them. Just as a ray of light, falling upon different objects, reflects the primary colors of the spectrum, so the best and highest inspirations of humanity are shaded into a play of moral colors, in direct relation with the mental attributes from which their views and actions are reflected. Whether the reasons for this are inherent, belonging to the structural or anatomical character, or whether they proceed from different varieties of the moral perceptions, may safely be left to the theologian, or remanded to the arena of the metaphysician.

*We* deal with the fact; and from this it would appear, that a code of written rules, prescribing so far as may be, the nature of the obligations, duties and relations of physicians, is as necessary to the corpus medical, as to the body politic. By such means, and such means only, can the intricate questions arising out of the gored ox, be satisfactorily settled.

This proposition, then, being conceded as an abstract or theoretical verity, and the code in general being established, perplexing problems necessarily arise in its practical operations and executive administering. It would seem impossible to make laws covering every shade of wrong-doing, from the guarded insinuation, to the flagrancy of the overt act. No legislative tribunal has ever attempted such a thing; and with all the written statutory enactments pertaining to the administration of justice, personal rights would often be invaded, and the guilty go unpunished, were it not for the broad and benign principles of the common law.

The most that we, as medical men, can do, therefore, is to prescribe *general* rules relating to the intercourse of physicians, and establishing conventional tests of acceptable deportment in the prosecution of a double-featured calling; in the one aspect, a lofty, dignified, unselfish and humane vocation; in the other, bound by the accompanying fee to the haunts of trade, and nailed, like a base coin, to the counter. And just here it is that the whole difficulties of the case appear. In the aspect of scholar and philanthropist, exemplary and magnanimous professional conduct follows by natural sequence; in the garb of business, the tricks of trade are as irrepressible as Banquo's ghost, which would not down. Fortunate is it for the general welfare, that there are practitioners, and many of them, who without leading an ideal life, are sufficiently great to subordinate the latter to the former; or perhaps, more accurately, to run the machinery of the business with a "governor" constituted of the higher and nobler elements of the calling.

Hence, in the present nature of the case, it would seem impossible to accomplish more than as above indicated. We have not the machinery of courts, with judge, prosecutor, advocate and jury, backed not only by written laws, but rendered infallible by principles of equity; and all past attempts at their constitution, in form or essence, have been failures of necessity. The code must stand as a moral obligation, and the details of every-day action under it, must be remitted to the casuistic sense of each individual member of the profession. Flagrant acts of empyricism may be taken cognizance of, and punished, of course; but the refined charlatanism of our day—that of which we most complain—scorns all vulgar methods, and delights in the art which accomplishes an end with the skill of the *prestidigitateur*, who in the pride of his achievements, assures us that now it's visible, and now it isn't.

It is no grateful task to assume the severe character of a censor, or to arrogate to one's self the attributes of an exemplar. But an old disease has broken out with renewed intensity, and fastened itself upon the modern profession of medicine; and it is neither the part of wisdom nor of manhood to disavow it, or shirk the encounter. Upon the contrary, it is no less a duty to ourselves, than to the general public, whose well-being in one sense, we are supposed to hold in keeping, not by a mere medical fiction, but in reality, that we meet the question squarely at all times, and determinedly apply ourselves to its best solution. By such means only can we hope in this, as in all other human problems, "Out of the nettle, danger, to pluck the flower, safety."

Here we tread upon perilous ground, and it is essential at the outstart, to define our positions clearly, that they may be in no danger of misinterpretation. What, then, is this disease of the modern body medical? It certainly does not consist in the circumstance of a certain number of men living in open violation of a code of ethics adopted by the American Medical Association. This would be but a narrow view of the case, the true cause of which stretches its roots far deeper than questions of mere medical polity, and grows from the subsoil upon which rest the very foundations of our modern medical temple. Perhaps there has never been a time when there were fewer examples of open or penal infraction of the ethics of the "regular" profession, (a distasteful expression, but apparently necessary as a generic term) than in our own day. As satellites of a central luminary, representing, as we believe, the sum of our present knowledge of medical truth, we revolve around the code of the National Association, within a very clean-cut orbit, and he who swerves but a degree beyond it, soon goes whirling into the regions of medical fallacy and pretension.

Our parallel ceases here, however, and there can be adduced no further similitude with the systems of worlds which travel through illimitable space upon celestial time-tables. Our orbit is a sort of Rubicon, beyond which we must not go, but *within* which many deem themselves privileged to take cross-cuts and royal roads; and in pursuing their erratic flight, to occult as many of their fellow twinklers as possible.

To treat our case upon principles of medical logic, it would be appropriate to regard the symptoms of the disease as the premises of a syllogism, and deduce the cause as the inevitable conclusion. The former, then, in a few words, may be said to consist in subordinating the higher elements of the profession to its baser features as a trade or money-getting art. Your correspondent knows not how better to formulate it in a single, dogmatic sentence.

It would be an unpalatable, as well as ungracious task to cover by narration, the arts, tricks, devices and methods so energetically resorted to by many physicians, of all grades and of every school and *ism*, whereby all possible soils are "worked" that they may bear a return to the industrious cultivator. From the doctor who runs the ward meetings, hoping to reap the political places, to him who gets invited out to society gatherings and displays his æsthetic cultivation by select readings from the poets, there exist all shades of art in the business; running through every color, embracing every *pose*, depicting every possible perspective, and representing the crudities of the 'prentice hand, as well as the finish of the master. "All roads lead to Rome," and through whatever devious and narrow channels these efforts run, they but represent the acts which

> "Crook the pregnant hinges of the knee,
> That thrift may follow fawning."

Now, were such practices confined to the legions of pretenders, sailing under the flag of an *ism*, honest men might treat them with the scorn they deserve; but it is better to out with the ugly truth and confess they are not. Let no man say that this is an overdrawn statement. The proof is too accessible for successful denial. Far be it from the purpose of the writer to be hypercritical or harsh in judgment, and above all, to fail in a proper tribute to those who love their profession for its inherent worth, and who regard its pecuniary emoluments as did John Hunter, when, upon rising from a protracted dissection, he was wont to throw down his scalpel and exclaim, "I shall have to go and earn that miserable guinea."

But within the minds of many, no doubt, who will read these lines, there are not a few modest and worthy medical men beginning to ask themselves some pretty hard questions upon this subject, and among others, perhaps something like this: "Wherein lies the difference between *legitimate* advertising by means of such reported cases and lectures, startling operations, etc., as are *specially prepared* for the profane eye and ear, and sure to find their way, by allusion or quotation, to the secular journals, and the more open one of boasting in public of cures performed, and playing fantastic tricks before a credulous audience? Or, perhaps, the subtle distinction between appending recommendations to Lacto-Peptine, a preparation proposing to relieve the stomach of any agency in the digestive process; or mayhap, to a mineral water differing by inappreciable fractions of composition from some other water, either of which may be as important in a therapeutic, as was the urine of Sir John Falstaff in a diagnostic point of view, as appears from the following:

*Falstaff.* "Sirrah, you giant, what says the doctor to my water?"

*Page.* "He said, sir, the water itself was a good healthy water; but for the party that owned it, he might have more diseases than he knew for."

What is the difference, it may be asked, between such recommendations, and certificates to the astounding efficacy of a patent medicine!

Let us be just, as well as temperate in expression; but let us not be too punctilious, or too timid to call things by their proper names.

The experience of every medical man of this day affords him personal knowledge of the many oblique lines leading to professional reputation, and pecuniary success; and true must he be to the dignity and honor of his calling, if, as a witness to the wild steeple-chase of success, and when, as it would almost seem, madness rules the hour, he can preserve his equanimity and bide the time for worth and merit to come to the stand.

If this philippic be grounded in truth and justice, and the symptoms of our sickness have been faithfully portrayed, what are the causes, it must be asked, lying behind such a distemper?

They are many in detail, and as per consequence, although few in substance and essence. Among the more potent of these, is the over-production of doctors, and the promiscuous adoption of a profession crowded to such an excess as to render competition for patronage a matter of business with many; and forcing some, in the struggle for sustenance, into the position of the poison-vending druggist in the play, when exclaiming

"My poverty, but not my will, consents."

The writer of this article, while an editor of a medical journal in a neighboring State, several years since, had occasion to discuss the question of medical education in all its diversified bearings, and ventured then to predict many of the evils which are now upon us. It was easy to foresee the result of the system of establishing medical schools at every cross-roads, and turning out graduated physicians, utterly ungrounded even in the elements of the profession. This milling process, however, has not been confined to the typical cross-roads; but when a professor in a certain metropolitan school asked a candidate for a degree, as the only question he had to propound, "How to make a mint-julep?" he simply stated the amount bid by his institution for popularity and patronage.

And when speaking thus of the "regular" profession (*sic passim*), we must not forget the accumulative sum of the *regular* quacks. They have their schools, too—their "courses" of study—their fees, and their diplomas.

Until medicine becomes (if it ever shall) an exact science, and like astronomy, mathematics, etc., there be but one arithmetical statement of the problems of health and disease, there must, of necessity, be all sorts of theories and all varieties of practices—much of the first wildly false, and much of the second intensely dishonest.

Of course, there must be a remedy for all these things, and possibly it may be found in advance of the millenium. But when it shall come to the world, it must come by agitation; it must come by the education of the people, and it must come through the schools and the masses of the profession. In some foreign countries, whose peoples are not supposed to be so enlightened as those of the "Great Republic," there are—

curiously enough—better methods for the protection of the population from empyricism than obtain with us.

If it were possible to make a medical diploma represent genuine qualification for the practice of medicine, (and some of our schools are undoubtedly laboring toward that end) and in case of those professing to dissent from the therapeutic theories of the "old school," to bear evidence of a thorough knowledge of such branches as are insusceptible of double interpretation—anatomy, chemistry, botany, mineralogy, obstetrics, operative surgery, etc., etc.—a long step would have been taken toward remedying present evils. How much of this, however, must be done by the State; how much by the schools of medicine, and how much by the profession at large, are interesting questions, admitting of wide discussion.

The writer has his own views upon the subject, and may be tempted to express them upon a future occasion. At present, his remarks have so far exceeded the limits which he designed for them, that he almost deems an apology necessary for their length, though he considers none requisite for their apparent warmth and energy. He believes that it has become too largely the custom to speak of the things herein treated, in guarded sentences, and almost with bated breath, as though their mere mention involved a lack of dignity, or a dangerous confession. Charity, it has been said, is the greatest of all virtues, but the time often comes when it is a mistaken policy, and an absolute injustice to the general welfare. Respectfully,

C. A. Logan.

99 Ashland Avenue.

